# Pytalism from Amalgam Fillings

**Published:** 1872-09

**Authors:** H. Scott

**Affiliations:** Lancaster, Ohio


### Pytalism from Amalgam Fillings.

BY H. SCOTT.

Upon this subject much has been written and said. The following is such a case that I feel I should be chargable with culpable neglect of duty if I did not report it.

Yesterday when I came to my door, returning from church, a gentleman and lady from the country drove up and asked if I was the dentist. I bid them alight, when the gentleman said he would first inquire if it would be worth while; and went on to say that his wife had some teeth filled two weeks ago, and had been sick ever since, and wanted the fillings taken out; and added, that they had been to the dentist who filled them, and he had refused to remove them. I replied that I could give no opinion until I had seen the mouth.

The lady had no sooner taken her position in the chair than the familar mercurial fetor reached my sense, and I enquired if she had been taking medicine lately; she said she had not, except some suger-coated pills (Ayres), "day before yesterday." I at once commenced an examination, and found eight amalgam fillings, four below and four above. The metal was rough on the surface, and part of the large amalgam fillings had been washed away. Upon touching them with a probe, I found it passed into them readily, causing the metal to crumble away in fragments, though two weeks had elapsed since it was put there. I asked what kind of taste she had in her mouth, and she said it tasted like brass, or some kind of metal. I next examined the circulation, and found the rapid pulse invarably found in pytalism. The patient said she had head-ache; impaired appetite; with more or less fever every day, and felt sick generally; and that her teeth were extremely sensitive to variations of temperature. She said, in answer to other inquiries, that she was well in every way when she went to the dentist, and that the sickness came on three or four days afterward. I asked her to state how the dentist prepared the fillings, and how long he was about it; and she replied that he mixed the "stuff" on some kind of a flat thing, from which he removed it to her mouth; and that he did it all in less than an hour, and charged her sixteen dollars.

I felt the responsibility that the case involved to me, and called an experienced physician, without intimating to him what I wanted. He had no sooner came to the patient's side than he said: "The lady is salivated." I then called his attention to the amalgam fillings, and explained that the mercury had not been squeezed from the mass, when he remarked that the mercury had been absorbed, producing its constitutional impression, beyond all possible doubt. It was the excess of mercury which in this case did the mischief. And I affirm that I would risk five times the same amount of amalgam in five thousand mouths, and fear nothing, provided the amalgam had been properly prepared before introducing it.

The fillings were all removed inside of three minutes, with a common steel excavator, they offering no resistance whatever. I am prepared for the responsibilities of the case.

LANCASTER, Ohio.<br>

Sept. 2, 1872.

